# Spatial clusters of violent deaths in a newly urbanized region of Brazil: highlighting the social disparities

**DOI:** 10.1186/1476-072X-8-66

**Published:** 2009-11-27

**Authors:** Ruth Minamisava, Simonne S Nouer, Otaliba L de Morais Neto, Lícia Kamila Melo, Ana Lucia SS Andrade

**Affiliations:** 1 School of Nursing, Federal University of Goiás, Faculdade de Enfermagem, Rua 227 s/n Setor Leste Universitário, Goiânia, Goiás, Brazil; 2 Department of Preventive Medicine, The University of Tennessee Health Science Centerd, 66 N Pauline, 38163, Memphis, USA; 3 Department of Analysis of Health, Secretariat of Surveillance on Health, Ministry of Health, Esplanada dos Ministérios, bloco G, Edifício Sede, Distrito Federal, Brasília, Brazil.; 4 Secretariat of Health of Goiânia Municipality, Praça Boaventura, n°149, Setor Leste Vila Nova, Goiânia, Goiás, Brazil; 5 Department of Community Health, Federal University of Goiás, Goiânia, Instituto de Patologia Tropical e Saúde Pública. Rua 235, esquina 1a. Avenida, Setor Leste Universitário, Goiânia, Goiás, Brazil.

## Abstract

**Background:**

Deaths due to homicides and traffic accidents among youth are a public health issue worldwide. Studies of the complex network of cause and effect on this topic point to both poverty and health inequalities. Different investigational approaches to intentional and unintentional deaths combined with socioeconomic variables can help create a better understanding of the association between violence and socioeconomic conditions. This study analyzed the spatial distribution and potential clusters of risk for intentional and unintentional deaths among youths aged 15-24 years in Goiânia, a newly urbanized city in central Brazil.

**Methods:**

Death data and residential addresses were extracted from the national Mortality Information System and validated by household visits. To detect all potential cases, we prospectively investigated every death classified as a transport accident, assault, legal intervention, intentional self-harm, unknown underlying cause, and undetermined intent according to the ICD-10.

The Geographical Information System was used to plot residential addresses, and cases were interactively geocoded to the residential address level using a digital map of the municipality. Spatial scan statistic was applied (Poisson model) to identify clusters of census tracts with high mortality due to intentional injuries and traffic accidents. The socioeconomic variables obtained using census data were compared between the most likely cluster and other areas of the municipality.

**Results:**

The most violent deaths among young people were due to intentional injuries. Between August 2005 and August 2006, 145 addresses for cases of intentional injuries and traffic accidents were located and geocoded. No significant clusters for deaths due to traffic accidents were found within the municipality. One significant cluster (RR = 4.65; p = 0.029) composed of 14 cases of intentional deaths, mostly homicides, was detected in an emergent, populated, and very poor area on the outskirts of the town. This cluster had a significantly higher proportion of people with the lowest educational status, lowest income, and poor housing conditions in comparison to the remainder of the municipality.

**Conclusion:**

Our findings highlight the link between social inequalities and intentional deaths, clearly showing the need for urgent social interventions to reduce violence and premature mortality.

## Background

In recent years, deaths due to external causes have become an important public health issue, particularly in low- and middle-income countries. Injury is a leading cause of mortality in young people, and the burden of mortality from injuries due to road traffic accidents and violence is expected to increase worldwide over the next few decades [1,2]. Deaths from intentional injuries (e.g., homicides and legal interventions) and traffic accidents among youths may ensue from multiple factors related to the individual and their social background.

There are many theories regarding which socioeconomic conditions are related to violent mortality rates; however, the complex network of factors that contribute to the cause-effect chain between violence and social conditions remains unclear. For instance, studies have suggested that most violent deaths are linked to inequalities in income distribution [3,4], social cohesion and social capital [5,6], social trust and group membership [7], and levels of investments in education, health care, and housing [8].

Some studies have emphasized that socioeconomic levels can affect health and mortality outcomes when spatial analysis is carried out in small geographic areas, such as in census tracts. Larger levels of data aggregation, e.g., states or countries, have been important as they point out socioeconomic inequalities in health and mortality [9,10].

Geographic Information Systems (GIS) are an emerging technology in injury research, and are primarily used to analyze the spatial distribution of injuries, to map trauma services, and to assess target risk areas for resource allocation. Geo-spatial data regarding deaths due to intentional and unintentional injuries combined with social-economic variables have been added to injury prevention policies to help guide cost effective interventions [11-13]. In Brazil, studies using GIS have shown a relationship between low socioeconomic status and homicide violence [14,15]; however, these studies analyzed large geographic areas such as states, counties, or other broad administrative regions, potentially hiding dramatic differences across areas [16].

There are large inequalities in health between and within countries, and Brazil is recognized as a country with large social and health inequalities, some of which are potentially avoidable [17,18]. Brazil has the highest burden of intentional injuries on the American continent [19]. Injuries account for 90% of deaths, and homicides rank as the first cause, followed by road traffic accidents, for young people aged 15 to 24 years. This age group represents about 20% of the Brazilian population [20].

Although the overall mortality attributed to traffic injuries has declined since the approval of the Brazilian traffic law (end of the 90's), accidents and deaths involving motorcyclists have sharply increased in recent statistics [21], leading the government to set the prevention of violence and traffic accidents as a high priority, especially in urban settings [22]. Additionally, recent data from the World Health Organization place Brazil as having the world's highest homicide rates among individuals aged 0-24 years [23]. The growth in firearm-related homicides [24] has justified the introduction of legislation limiting the carrying of firearms [25]. In addition, a voluntary buyback gun campaign (exchange of guns for money) has been put in place. Soon after these interventions, a decline of 8.2% in the gun-homicide rate was achieved from 2003 to 2005 in Brazil [26]; however, in some cities, such as Goiânia, which has been undergoing a period of accelerated population growth over the last ten years, homicide rates among adolescents and young adults have increased from 49.43/100,000 in 2004 to 53.79/100,000 in 2005 [21]. This finding has prompted us to investigate the spatial distribution of intentional and unintentional deaths among youths using census tracts as the spatial unit of analysis as a complement to formal epidemiological analysis. Our main purpose has been to detect potential clusters of risk for timely interventions.

## Methods

### Study area

**Figure 1:**
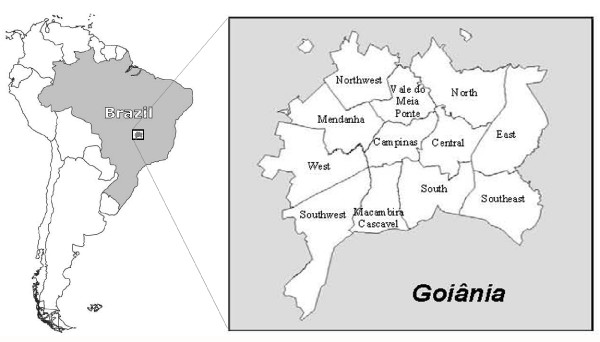
**Location of study area showing the municipality of Goiânia with the 12 health districts**.

**Figure 2:**
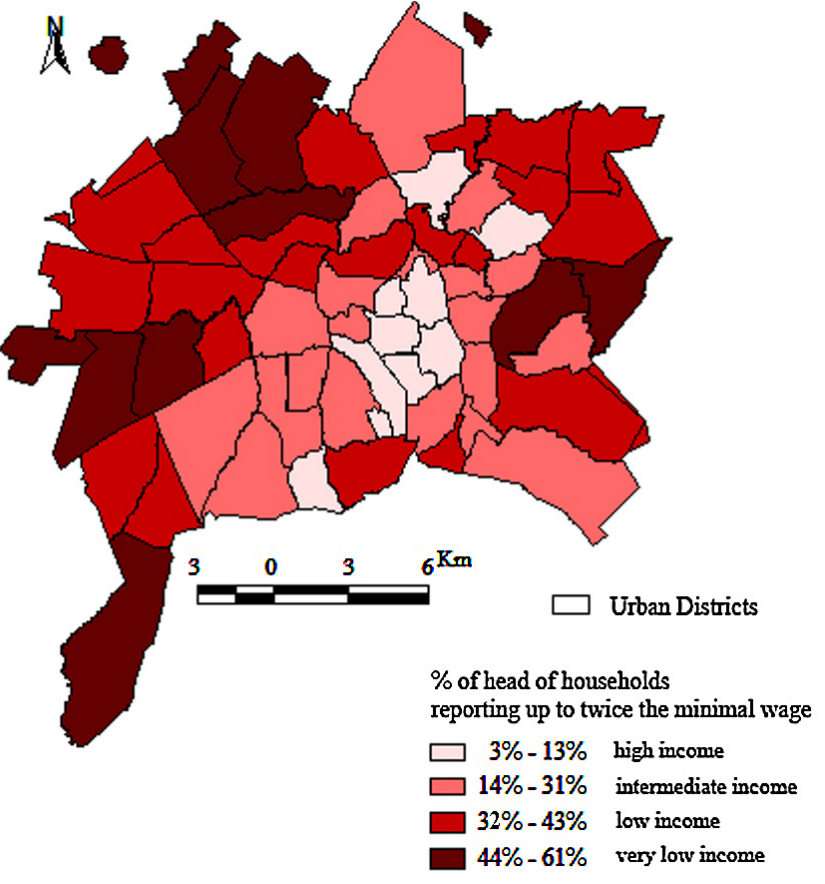
**Socioeconomic status by districts according to head of household's income**. Municipality of Goiânia, Goias State, Brazil. Source: Andrade et al. Cad Saude Publica 2004, 20(2):411-421.

The study was conducted in the municipality of Goiânia (1,201,007 inhabitants), capital of the Goiás State, located in the center-west region of Brazil (Figure 1). The municipality has an urbanized area of 739,492 square kilometers. In Brazil, the structure of the health sector is defined by the Unified Health System, and is based on principles of universal health care coverage. The Health District is the geographical and administrative division that manages public health services. The municipality has 12 Health Districts that have boundaries that are geographically delimited according to the intra-urban flow of people. High-income districts are located in the extreme south of the "Central Health District" and in a central-north area of the "South Health District," while there are concentric circles of escalating poverty towards the outskirts. Four very low socioeconomic areas can be distinguished in the peripheral districts (Figure 2). The northwest and southwest areas are characterized by new settlements. The west and east areas encompass a more well-established community [27].

### Data sources

Death data, including residential addresses, were extracted from the national Mortality Information System (MIS), which is fed by Death Certificates generated in Goiânia. Eligibility criteria for the study were: deaths due to transport accidents, assaults, and legal interventions that occurred from August 5, 2005, through August 4, 2006, among individuals aged 15-24 years. To detect all potential cases, we also prospectively investigated every death classified as a transport accident (codes V.01-V.99), assault (codes X.85-Y.09), legal intervention (code Y.35), intentional self-harm (codes X.60-X.84), unknown underlying cause (codes R.95-99), and undetermined intent (codes Y.10-34), according to the International Statistical Classification of Diseases and Related Health Problems, 10th Revision [28]. Household visits were conducted for all potential eligible cases to validate the underlying cause of death, birth date, and address.

The at-risk population comprises youth aged 15-24, which is estimated to be 264,005 individuals living within the boundaries of the municipality. Goiânia is divided into 1,066 census tracts, and each encompasses approximately 300 dwellings with a mean of 1,018 inhabitants [29]. The population at risk and socioeconomic data at the census tract level were downloaded from the Brazilian Census 2000 website ftp://ftp.ibge.gov.br/Censos/Censo_Demografico_2000/Dados_do_Universo/Agregado_por_Setores_Censitarios. We selected eight socioeconomic census variables obtained from 100% of the Brazilian housing units (race is not included) by household visits based on their adequacy to characterize socioeconomic status [4,30-32]: (a) number of households with six or more inhabitants, (b) number of heads of household with < 8 years of formal education, (c) number of heads of household with ≥ 12 years of formal education, (d) number of heads of household with monthly incomes < 2 minimum wages, (e) number of heads of household with monthly incomes > 20 minimum wages, (f) number of households with garbage collection and with a public water supply system, (g) number of owned households, and (h) head of household average monthly income. We used education variables with cutoff points at < 8 and ≥ 12 years of formal education. In Brazil, elementary school is mandatory and lasts for 8 years; people with 12 years or more of schooling attended at least one year of college. Head of household monthly income < 2 minimum wages has been a common and arbitrary cutoff used for research purposes in Brazil. During the period of study, the monthly minimum wage ranged from U$ 110.00 to U$ 130.00.

### Geocoding process

Cases were interactively geocoded to the residential address level using a digital map dataset (SIGGO v.2 software) provided by the Data Processing Company of the Municipality of Goiânia. The digital map displays blocks, areas, lots, streets, and Cartesian coordinates. We generated a map layer using GIS software from ESRI (Environmental Systems Research Institute, Inc., Redlands, United States) based on individual points that were spatially joined to the census tract shape. For analysis purposes, deaths due to assaults and legal interventions were pooled into the intentional injuries category. Unintentional injury was represented by transport accidents. We created individual shape files for deaths due to intentional and unintentional injuries. The spatial unit of analysis was the census tract, but to better visualize the cluster locations, we also used the Health Districts' shape file.

### Statistical analysis

SaTScan™ software (v7.0.3) [33] was used for spatial analysis. The spatial scan statistic was based on a Poisson model to identify clusters of census tracts with high mortality [34] due to intentional injuries and traffic accidents, using population data from 2000 at the census tract level [29]. Because the boundaries of the census tract slightly exceed the borders of the Health Districts, the display of census tract polygons overlapped with the Health District layer. Five census tracts with extremely low values for youth population (≤ 45) were identified as outliers and, hence, were excluded from the analysis. The total population in the excluded census tracts was 135 inhabitants, which corresponds to 0.06% of the population at risk. We also excluded from the analysis cases for which addresses could not be found and geocoded.

The scan statistic sets a series of circular windows of varying radii over the study area. For our study, the radius of each circle was set to increase continuously to include up to 50% of the total population at risk. Since the spatial scan statistic is used to detect clusters in a point process, the maximum number of points in each window was recorded and compared to its distribution. Each circle encloses different sets of census tracts and represents a potential cluster. Under the null hypothesis that injury deaths would be expected from a purely random (Poisson) process, the number of cases in each census tract is proportional to the size of the population [35]. The likelihood ratio and p value were obtained by Monte Carlo simulations using 999 replications. The circle with the maximum likelihood represents the most likely cluster. Secondary, non-overlapping clusters are found by excluding points of the most likely cluster from the dataset and replicating the above procedure. SaTScan^® ^reports the most likely cluster and secondary clusters in its output, along with the corresponding relative risks and p values (one-tailed). Clusters with high mortality were displayed using ArcView software v.3.2. The comparison of socioeconomic characteristics of the most likely cluster variables among census tract groups was performed using a chi-squared test to compare differences in proportions, while differences between means were assessed with a *t*-test. Statistical significance was set at the 0.05 probability level.

### Ethics

The study protocol was approved by the Research Ethics Committee of the Emergency Hospital of Goiânia. Informed consent was obtained from all legal representatives or proxy informants of the victims.

## Results

**Figure 3:**
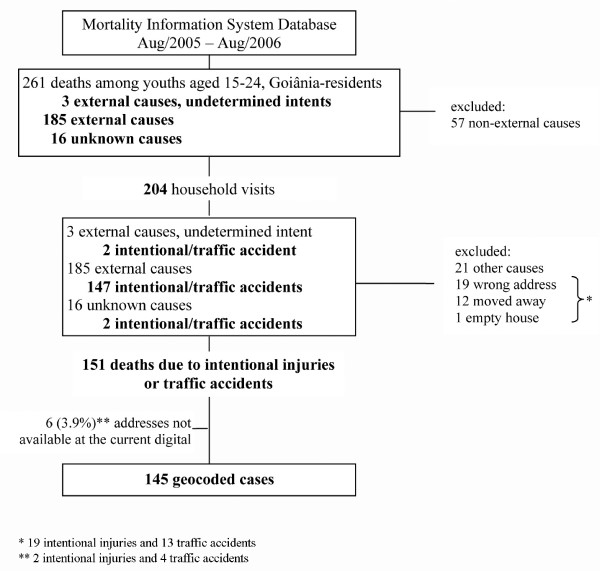
**Flow chart for identification of violent deaths in youths**. Goiânia, Brazil, Aug/2005-Aug/2006.

During the study period, a total of 261 deaths among Goiânia-resident youths aged 15-24 years were reported by the MIS. Of those, 204 were considered potential cases, and thus, their households were visited (Figure 3). We were able to validate 151 Goiânia resident's cases as deaths resulting from intentional injuries or from traffic accidents. After household visits, changes in the ascertainment of death causes were as follows: (i) two undetermined intent cases changed to one traffic accident and one assault by handgun discharge; (ii) one traffic accident changed to assault by the crashing of a motor vehicle; (iii) nine assaults by handgun discharge changed to nine legal interventions involving firearm discharge; and (iv) two unknown death cases changed to one traffic accident and one assault by handgun discharge. Overall, a 90.7% agreement between the MIS information and the household interviews was achieved, and no difference in age was noted between these two data sources. We excluded 21 other causes of death, including mostly suicide, unknown cause, infectious diseases, neoplasm, and complications of surgical care. A total of 32 addresses could not be validated. Thus, among the 19 deaths with incorrect addresses, 12 were due to intentional injuries cases and 7 were due to traffic accidents. Additionally, 12 households had moved away (7 intentional injuries and 5 traffic accident deaths).

During the process of georeferencing, we could not find addresses for six (3.9%) cases on the digital map dataset (SIGGO software, two intentional injuries and four traffic accidents) since they were located in new settlements and, hence, were not available in the current digital map. Of the 145 geocoded cases, the mean age was 20.2 years (standard deviation = 2.66), and 138 (95.2%) were males. Most of the deaths (66%) were caused by intentional injuries (Table 1). No significant difference was found between the mean age of cases for traffic accident and intentional injuries. About 60% of cases were between 20 and 24 years old.

**Table 1:** Classification of deaths due to homicides and traffic accidents among individuals aged 15-24 years. Goiânia, Brazil, Aug/2005-Aug/2006.

Violent deaths	15-19 years		20-24 years		Total
			
	N	(%)	N	(%)	
**Intentional injuries (N = 96)**					
Assault by handgun discharge	28	(47.5)	44	(51.1)	72
Assault by sharp object	6	(10.2)	6	(7.0)	12
Assault by other specified and unspecified means	1	(1.7)	2	(2.3)	3
Legal intervention	6	(10.2)	3	(3.5)	9
**Traffic accidents (N = 49)**					
Motorcycle rider	15	(25.4)	22	(25.6)	37
Car occupant	3	(5.1)	2	(2.3)	5
Pedal cyclist	0	(0.0)	3	(3.5)	3
Pedestrian	0	(0.0)	3	(3.5)	3
Other and unspecified transport accidents	0	(0.0)	1	(1.2)	1

Total	59	(100.0)	86	(100.0)	145

**Figure 4:**
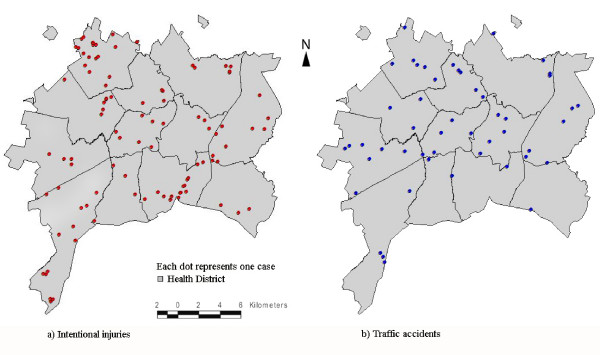
**Point pattern distribution of deaths due to intentional injuries and traffic accidents among youths aged 15-24 years**. Goiânia, Brazil, Aug/2005-Aug/2006.

**Figure 5:**
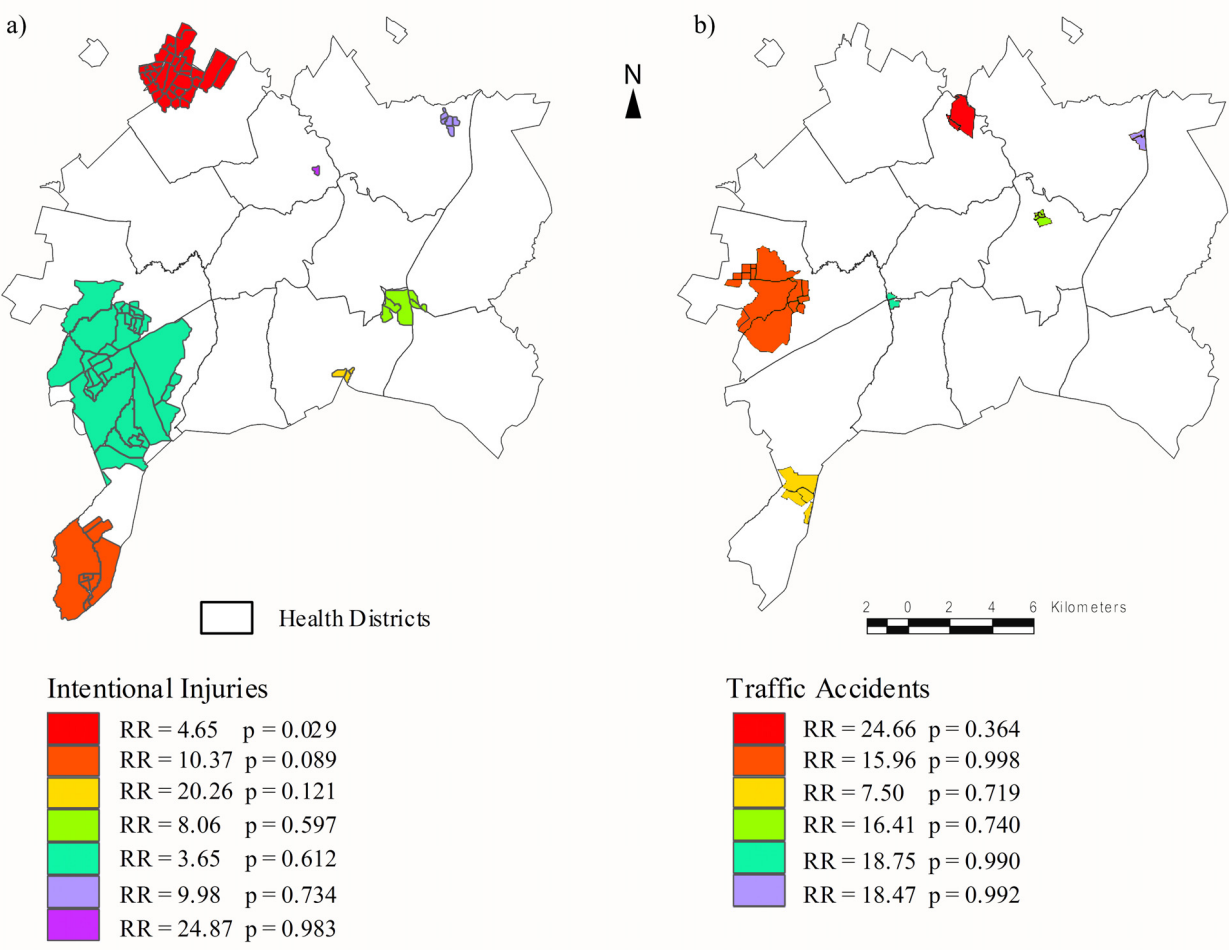
**Risk areas for deaths due to intentional injuries and traffic accidents among youths aged 15-24 years**. Goiânia, Brazil, Aug/2005-Aug/2006.

Figure 4 shows the point pattern distribution of residences for the youths according to injury classification. By visual inspection, the distribution of deaths from traffic accidents seems to occur homogeneously within the city, while deaths from intentional injuries are distributed in the peripheral areas of the municipality. After applying the spatial scan statistic for intentional injuries, a geographic area located in the northwest part of the "Northwest Health District" of Goiânia was identified as the most likely cluster (RR = 4.65; p = 0.029). This cluster encompassed 34 adjoining census tracts located in the outskirts of the city (Figure 5a), and another 6 non-significant secondary clusters were identified (Table 2). Traffic accident deaths were randomly distributed within the municipality with no significant cluster detected by the scan statistic (Figure 5b).

**Table 2:** Characteristics of spatial clusters* of intentional deaths among individuals aged 15-24 years. Goiânia, Brazil, Aug/2005-Aug/2006.

Cluster type	No. of census tracts	Observed cases	Expected cases	Population	Relative Risk	P value
Most Likely Cluster	34	14	3.4	8,445	4.65	0.029
Secondary cluster 1	9	6	0.61	1,525	10.36	0.089
Secondary cluster 2	3	4	0.21	511	20.26	0.121
Secondary cluster 3	6	5	0.65	1,616	8.06	0.597
Secondary cluster 4	34	11	3.29	8,181	3.64	0.612
Secondary cluster 5	5	4	0.42	1,035	9.98	0.734
Secondary cluster 6	1	2	0.08	204	24.97	0.983

*Spatial scan statistic.

When comparing the most likely cluster to the remain clusters and to the rest of the municipality (Table 3), the most likely cluster had significantly higher proportions of people with the lowest educational status, lowest income, and poor housing conditions (p < 0.001); however, it also presented a significantly higher proportion of owned households.

**Table 3:** Demographic characteristics of census tract clusters of homicides and the remainder of the municipality according to variables available at census. Goiânia, Brazil.

Census Variables	Most likely cluster*		Remainder of the clusters**		Remainder of the municipality***	
			
	N	(%)	N	(%)	N	(%)
Heads of household with < 8 years of formal education	8918	81.26	10468	60.22^§^	126852	44.78^§^
Heads of household with ≥ 12 years of formal education	72	0.66	924	5.32^§^	47270	16.69^§^
Households with six or more inhabitants	1301	11.86	1734	9.98^§^	23094	8.15^§^
Households with garbage collection and with a public water supply system	9618	87.64	8548	49.17^§^	254240	89.75^§^
Owned households	8696	79.24	9720	55.92^§^	153854	54.31^§^
Heads of household with monthly incomes < 2 minimum wages	5144	46.87	5332	30.67^§^	56547	19.96^§^
Heads of household with monthly incomes > 20 minimum wages	14	0.13	225	1.29^§^	20448	7.22^§^
Monthly income of the heads of household (means, SD)	R$ 286.10 SD = R$41.52		R$ 611.33^† ^SD = R$638.60		R$ 1117.42^§ ^SD = R$1069.69	

* total = 10,974 heads of households

** total = 17,383 heads of households

*** total = 283,290 heads of households

§ Compared with the most likely cluster, p < 0.001

SD Standard deviation

† Compared with the most likely cluster, p = 0.04

## Discussion

This study disclosed different patterns of the spatial distribution of deaths when comparing intentional injuries and traffic accidents among youths. While no significant clusters for deaths due to traffic accidents were detected, two cluster areas for intentional deaths were found in two emergent and very poor areas on the outskirts of the town, namely, the northwest and southwest regions.

The significant cluster that exhibited a high risk area of homicides and legal interventions was spatially located at the periphery of the northwest region. Homeless individuals have illegally invaded both the northwest and southwest regions since the beginning of the 1980s. The settler population was entirely poor, unemployed or unskilled, young, and nomadic. In response to this social problem, the government has donated lots and building materials, set up healthcare services and police stations, covered the streets with asphalt, and lighted the streets. Thus, new settlements were created [36].

Unfortunately, these actions have contributed to differential residential segregation in these regions. The first settlement in the city was established in the northwest region and was followed by a high rate of population growth. As a consequence, poorer households have been increasingly pushed to the periphery of that region. Settlements in the southwest region are newer, having been established in the late 1990's, and the population is sparsely distributed along the region [36]. The northwest region contains the largest population at risk of violent death in comparison to all of the outskirt regions of the municipality, and this region is also a very low-income region according to a previous report [27]. Probably as a result of these reasons, the most likely cluster comprised a high proportion of owned households, more than 80% of heads of households had < 8 years of formal education, and almost half of heads of households had a monthly income of less than two minimum wages.

Several studies have shown that various social conditions are associated with homicide [4,6,37-40]. Similarly, our results exhibit high mortality due to intentional injuries in a unique cluster that encompasses a deprived area of the city. This cluster was precisely found in an area that included features in addition to low income: high population growth, high population density, fast urbanization, artificial process of settlement, social segregation, and history of expressive social conflicts and political issues. In our study, the geographic differences in homicide rate were associated with socially disadvantaged groups, such as those with low income, schooling, and social segregation. Thus, deprived socioeconomic conditions seem to be one of the factors that may yield social and health inequalities. It has been suggested by some Latin American authors [4,15,41] that social disadvantages play a crucial role in homicide rates, as a trigger for homicides.

We did not find evidence of areas of significant risk of death due to traffic accidents, as others authors have [42,43]. Overall, youths who died due to transport accidents were motorcyclists. While no area of significant risk was found, in 2003, Goiânia exhibited the highest traffic accident-related mortality among all Brazilian state capitals [44]. Besides the fact that many motorcycles in Goiânia are ridden by disadvantaged young people and/or motorcycle-delivery and motorcycle-taxi drivers, another concern is that most of these drivers are inexperienced drivers. The random geographic distribution of these deaths may be explained by a recently growing middle class that has never even ridden in motor vehicles [45]. Motorcycles are a relatively inexpensive means of transportation for disadvantaged people and may also be recreational vehicles for wealthy people.

Because we used a spatial unit of analysis, which is the lowest level of spatial disaggregation available in the census data, we had a greater chance of using homogeneous intra-urban areas and unmasking the complex network of factors related to intentional deaths of youths in the context of the most likely cluster. Nevertheless, the lack of significance in the secondary clusters may be overlooked due to the use of small areas [46]. Over and above an ecological bias, unsuccessful attempts to find families for interviews is a potential source of downward bias; thus, the mortality rates presented here tend to be conservative.

The limitations of this study should be mentioned. We were not able to find the residences of 12 intentional injury cases and 7 traffic accidents during the household visits. Therefore, the possibility of bias due to a lack of address information could be a matter of concern leading to a distortion of cluster detection or even cluster location. Some cases lacked necessary address information and could not be address-matched to any zip code of the municipality of Goiânia. In fact, these missing addresses partially matched related zip codes of the surrounding counties. It would be desirable to analyze other variables related to violence and traffic accidents, such as drug trafficking, alcohol and drug use, race, social capital, and social organization, but most of this information is unavailable at the census level in Brazil or, when available, lacks accuracy. It is well known that secondary databases often provide inaccurate mortality data in less developed regions; however, the database from the MIS for the center-west of Brazil represents an acceptable quality of cause-of-death data [47].

## Conclusion

This study exemplifies the usefulness of the Geographic Information System in guiding policy makers to tailor interventions towards risk areas for intentional deaths in a recently urbanized area. The most likely cluster identified can be the first place to reinforce actions for the prevention and control of injuries. Our findings suggest that intentional deaths are linked to social inequalities, highlighting the need for urgent social interventions to reduce violence and, consequently, to decrease premature death.

## Competing interests

The authors declare that they have no competing interests.

## Authors' contributions

RM, ALA, SSN, and OLMN conceived the idea and the design of the study. LKM, RM, and SSN conducted most of the data preparation and the spatial analysis. LKM, OLMN, SSN, ALA, and RM interpreted the data. RM and ALA worked on the first draft of the manuscript. All authors provided intellectual input, read, and approved the final version of the manuscript.

## Funding/Support

This study was supported by research grant # 505324/2004-0 from the Ministry of Science and Technology and the Brazilian Council for Scientific and Technological Development (CNPq).
